# Pregnancy Outcomes among Obese Pregnant Women with Hypothyroidism: Medical Record Review of a Single Tertiary Center in Saudi Arabia

**DOI:** 10.7759/cureus.6938

**Published:** 2020-02-10

**Authors:** Anas M Fallatah, Anhar Hasanain, Hussam Babatin, Khalid M Nassibi, Samaher Thigah, Hassan S Abduljabbar

**Affiliations:** 1 Medicine, King Abdulaziz University, Jeddah, SAU; 2 Obstetrics and Gynecology, King Abdulaziz University, Jeddah, SAU

**Keywords:** hypothyroidism, maternal, neonatal, pregnancy, obesity, bmi, outcomes

## Abstract

Background

Thyroid disorder is common among pregnant women. Hashimoto thyroiditis is the most common etiology of hypothyroidism among pregnant women. Many studies showed that hypothyroidism during pregnancy has been associated with negative outcomes for the mother and for child as well including miscarriage, intrauterine growth retardation, preterm delivery and cognitive impairment in the offspring.

Objectives

To assess the adverse maternal and neonatal outcome among hypothyroidism obese pregnant women.

Methods

This is a retrospective study conducted among obese pregnant women diagnosed with hypothyroidism attending King Abdulaziz University Hospital (KAUH), Jeddah, Saudi Arabia between January 1, 2013, and December 31, 2018. For analysis, we used (1) descriptive statistics, (2) Chi-square test, Pearson correlation, independent t-test, and one-way ANOVA to test the difference in thyroid stimulating hormone (TSH) levels and adverse pregnancy outcomes. A p-value of <0.05 is used to calculate statistical significance.

Results

A total of 9095 pregnant women had delivered in the last five years, 65 of these pregnant women had been diagnosed with hypothyroidism and 57 were enrolled in our study. Out of 65, 44 (77.2%) were Saudi, and 13 (22.8%) non-Saudis. Mean age at the time of delivery was 32.9 ± 5.6 years, while BMI means were 35.7 ± 4.6. A total of 35 (61.4%) were from class 1, 14 (26.2%) were from class 2 and eight (12.3%) were from class 3. Out of 57, 16 (28.1%) developed undesired antepartum outcomes, while 14 (21.5%) had postpartum outcomes. Preterm labor, gestational diabetes mellitus, and urinary tract infections were significantly associated with abnormal TSH levels (P < 0.05).

Conclusion

As demonstrated earlier, hypothyroidism during pregnancy leads to unfavorable outcomes. Therefore, screening for thyroid function tests in prenatal and antenatal periods is vital to avoid potential adverse outcomes.

## Introduction

Hypothyroidism is outlined by a vast clinical scope ranging from an apparent of myxedema, end-organ and multisystem failure to an apparently healthy or subclinical states with elevated level of thyroid stimulating hormone (TSH) associated with normal level of circulating free T4 (FT4) and T3 (FT3) [1,2]. In the developed countries, hypothyroidism is seen in 4-5% of the population [3]. Moreover, subclinical hypothyroidism is found to be similar with 4-5% in the population [4].

Thyroid disorder is common among pregnant women [5]. Around 2-3% of those women will end up with undiagnosed hypothyroidism according to population studies [6,7]. Of these two-thirds will be diagnosed as subclinical, which means an elevated level of TSH with a normal level of circulating free T4. On the other hand, overt hypothyroidism is found in 0.5% of those pregnant women. It is a state where TSH level is elevated coupled with a decreased level of free T4 [8]. Hashimoto thyroiditis is the most common etiology of hypothyroidism among pregnant women, which is an autoimmune disease that results in gradual destruction of thyroid tissue [1].

Many studies showed that hypothyroidism during pregnancy has been associated with negative outcomes for the mother and for the baby as well including miscarriage, intrauterine growth retardation, preterm delivery and cognitive impairment in the offspring [9]. Subclinical hypothyroidism in pregnancy is related with higher risk of recurrent miscarriage, intrauterine growth restriction, preterm birth, low birth weight, perinatal mortality and pre-eclampsia [10].

A study conducted in Lebanon showed that 17% of 920 pregnant women had hypothyroidism during pregnancy and showed associations with a history of previous miscarriage and morbid obesity during pregnancy [11].

Another recent multicenter study conducted locally in Riyadh, Saudi Arabia (SA) showed that the prevalence of subclinical hypothyroidism in pregnant women was 13% [12]. An epidemiological study from 11 cities in nine states of India showed that 13.13% of pregnant women have hypothyroidism (n = 388), using a cutoff TSH level of 4.5 μIU/ml [13]. Hence, it is important to recognize and establish thyroid function test profile as a baseline to monitor the levels during pregnancy to avoid these bad consequences during and after pregnancy.

Despite the aforementioned studies, none have been conducted in SA which urges us to make our research. This study aims to assess the adverse maternal and neonatal outcome among obese pregnant women diagnosed with hypothyroidism in King Abdulaziz University Hospital, Jeddah, Saudi Arabia.

## Materials and methods

This is a non-intervention retrospective record review study that was carried out over five years period from the first of January 2013 to the end of December 2018. The study included obese pregnant women diagnosed before their pregnancy with hypothyroidism, who are attending King Abdulaziz University Hospital (KAUH), Jeddah, KSA. The electronic medical file records were obtained from the hospital’s Medical Records Department. Subjects with missing electronic files and BMI less than 30 kg/m^2^ were excluded. Out of 1037 obese pregnant women, 65 patients were diagnosed with hypothyroidism, and 57 were involved in the study for further analysis. The study was approved by the Institutional Review Board and the Research Ethics Committee of KAUH. To establish the state of hypothyroidism regarding these patients, TSH was taken at the first obstetric clinic visit or preconception clinic. We used the following for the trimester-specific TSH levels during pregnancy: (1) 0.1 to 2.5 mIU/L for first trimester, 0.2 to 3.0 mIU/L for second trimester, and 0.3 to 3.0 mIU/L for the third trimester. Most of our cases presented during the prenatal or first trimmest visiting. Variables regarding demographics were taken into consideration such as: nationality, gender, age, height, weight, and body mass index (BMI). All of the adverse pregnancy outcomes were defined according to the American College of Obstetrics and Gynecology (ACOG) latest guidelines in 2013 [14].

Then we took variables regarding their morbidity during pregnancy such as: preeclampsia, gestational diabetes mellitus (GDM), pregnancy-induced hypertension (PTH), antiphospholipid syndrome (APS), impaired glucose tolerance test (GTT), pre-premature rupture of membrane (PPROM), premature rupture of membrane (PROM), preterm labor (PTL), induction of labor, mode of delivery (Spontaneous vaginal delivery (SVD) or cesarean section (CS)), type of CS (emergency or elective), failure to progress (FTP), cephalopelvic disproportion (CPD), rate of cesarean section, anemia, and urinary tract infection (UTI); also, antepartum hemorrhage (Abruptio Placenta, Placenta Previa, Placenta accreta and low lying placenta), maternal mortality, fetomaternal complications (oligohydramnios, polyhydramnios, placental hematoma, and cord prolapse). Other variables regarding postpartum period were: postpartum hemorrhage (PPH), endometritis, vaginal laceration, perianal laceration 1st, 2nd, 3rd degree, internal hemorrhoids, surgical site infection (SSI), and wound dehiscence. Moreover, neonatal outcomes were also included such as: gestational age (GA), birth weight, fetal sex, fetal presentation, Apgar score at one and five minutes, admission to neonatal intensive care unit (NICU), birth defects and injuries, intrauterine fetal demise (IUFD), stillbirth, and neonatal death.

Statistical Package for Social Sciences (SPSS; Release 23.0.0.0, IBM Corp., Armonk, NY) for Windows software was used for the analysis of data. The one-way ANOVA and independent t-test and correlation tests were used for comparison of quantitative variables. The chi-square test was used as a test of significance for comparison of categorical variables. P-value less than 0.05 was chosen as the level of statistical significance.

## Results

The aim of the study is to identify the potential effects hypothyroidism has on maternal and neonatal outcomes among obese pregnant women. As shown in Table 1, out of 57 hypothyroidism pregnant women, 44 (77.2%) were Saudi, the mean age was 32.9 ± 5.6, the mean BMI was 35.7 ± 4.5, where 35 (61.4%) were from class 1, 14 (24.6%) were from class 2 and eight (14%) were from class 3. The mean scores of gravidity and parity were 5.0 ± 2.0 and 3.0 ± 1.0, respectively, where all 57 cases were multigravida and multipara. All the maternal (100%) had comorbidity, 18 (27.7%) developed morbidity during their pregnancy, 14 (21.5%) had postpartum complications, and none reported as a mortality case. All the cases (100%) reported no family history of DM type 2, while one (1.5%) case reported family history of thyroid.

**Table 1:** Demographic data, medical and obstetric history among the sample SD: Standard deviation; TSH: Thyroid stimulating hormone; BMI: Body mass index; CS: Cesarean section; DM: Diabetes mellitus.

Variable		TSH Levels			Total	P-value
		Low (N = 2)	Normal (N = 42)	High (N = 13)		
Age	Mean (SD)	31.75 (5.83)	31.85 (5.78)	32.97 (5.61)	32.9 (5.6)	0.049
Height	Mean (SD)	156.21 (6.20)	155.83 (5.81)	156.32 (6.60)	155.1 (6.7)	
Weight	Mean (SD)	78.67 (6.97)	89.58 (7.08)	107.55 (12.39)	86.1 (13.5)	
BMI	Mean (SD)	32.20 (1.38)	36.89 (1.38)	44 (4.07)	35.7 (4.5)	0.019
Parity	Mean (SD)	6.0 (2.0)	3.0 (1.0)	3.0 (1.0)	3.0 (1.0)	0.024
Number of CS	Mean (SD)	4.0 (0.6)	2.3 (1.5)	2.0 (0.9)	2.0 (1.0)	
Gestational age	Mean (SD)	39.00 (1.41)	37.90 (2.26)	38.62 (1.69)	38.0 (2.1)	
Nationality	Saudi	1 (50.0%)	34 (81.0%)	9 (81.0%)	44 (77.2%)	
	Non-Saudi	1 (50.0%)	8 (19.0%)	4 (19.0%)	13 (22.8%)	
Parity	None	0 (0.0%)	0 (0.0%)	0 (0.0%)	0 (0.0%)	
	One	0 (0.0%)	13 (31.0%)	3 (28.6%)	16 (30.8%)	
	Two	0 (0.0%)	10 (23.8%)	3 (19.0%)	13 (25%)	
	More than three	2 (100.0%)	19 (19.0%)	7 (33.3%)	28 (53.8%)	
BMI Class	Class 1	2 (100.0%)	26 (61.9%)	7 (57.1%)	35 (61.4%)	
	Class 2	0 (0.0%)	10 (23.8%)	4 (33.3%)	14 (24.6%)	
	Class 3	0 (0.0%)	6 (14.3%)	2 (9.5%)	8 (14%)	
Family history of Thyroid disease		0 (0.0%)	0 (0.0%)	1 (4.8%)	1 (1.5%)	
Family history of DM 2		0 (0.0%)	0 (0.0%)	0 (0.0%)	0 (0.0%)	
Maternal mortality		0 (0.0%)	0 (0.0%)	0 (0.0%)	0 (0.0%)	

As demonstrated in Table 2, the result revealed that three (7.1%) cases had diabetes type 2, while there was only one case that had hypertension (HTN), diabetes type 1, polycystic ovarian syndrome (PCOS), and bronchial asthma (BA) equally. Regarding maternal morbidity, only three (5.3%) cases had preterm labor, and five (8.8%) reported induction of labor. On the other hand, seven (12.3%) cases had GDM, two (3.5%) had pregnancy-induced hypertension (PIH), and one (1.5%) had APS, FTP, and PPH. In addition, we found a significant difference (P = 0.035) among those with elevated TSH levels who were more bound to have preterm labor in opposite to the others. Not only that, we also noticed they were more susceptible to develop GDM.

**Table 2:** Maternal comorbidity, and morbidity among the sample TSH: Thyroid stimulating hormone; FT4: Free T4; HTN: Hypertension; PCOS: Polycystic ovarian syndrome; PIH: Pregnancy-induced hypertension; GDM: Gestational diabetes mellitus; APS: Antiphospholipid syndrome; FTP: Failure to progress.

Variable		TSH Levels			Total	P-value
		Low (N = 2)	Normal (N = 42)	High (N = 13)		
Maternal comorbidity	HTN	0 (0.0%)	1 (2.4%)	0 (0.0%)	1 (1.8%)	
	Diabetes mellitus type 1	0 (0.0%)	1 (2.4%)	0 (0.0%)	1 (1.8%)	
	Diabetes mellitus type 2	0 (0.0%)	3 (7.1%)	0 (0.0%)	3 (5.3%)	
	PCOS	0 (0.0%)	0 (0.0%)	1 (7.7%)	1 (1.8%)	
	Neurological disease	0 (0.0%)	1 (2.4%)	0 (0.0%)	1 (1.8%)	
	Bronchial asthma	0 (0.0%)	1 (2.4%)	0 (0.0%)	1 (1.8%)	
Maternal morbidity	PIH	0 (0.0%)	0 (0.0%)	2 (15.4%)	2 (3.5%)	0.030
	GDM	1 (50.0%)	2 (4.8%)	4 (30.8%)	7 (12.3%)	0.011
	APS	0 (0.0%)	0 (0.0%)	1 (7.7%)	1 (1.8%)	
	FTP	0 (0.0%)	0 (0.0%)	1 (7.7%)	1 (1.8%)	
	Postpartum hemorrhage	0 (0.0%)	1 (2.4%)	0 (0.0%)	1 (1.8%)	
	Preterm labor	0 (0.0%)	2 (4.8%)	1 (7.7%)	3 (5.3%)	0.035
	Induction of labor	0 (0.0%)	5 (11.9%)	0 (0.0%)	5 (8.8%)	

Here Table 3 shows us a total of four cases who had poor score at 1 minute while none at 5 minutes. The mean GA was 38.0 ± 2.1, where 48 (85.7%) reported full term, seven (12.5%) reported preterm and one (1.8%) reported post term. Not only that, we found that those with high levels of TSH were negatively correlated with lower gestational age (r = 0.003). Furthermore, the birth weight mean was 3.1 ± 0.5, where three (5.6%) were low, 47 (87.0%) were normal, and four (7.4%) were macrosomia. Also, we found that those with high levels of TSH were negatively correlated with lower birth weight neonates (r = 0.145). More than half of the newborns (33, 61.1%) were male and 21 (38.9%) were female. In addition, more than half (37, 64.9%) had cephalic presentation, while six (10.5%) had breech presentation. Fetal death was reported in one case only. NICU admission was reported in four (7.0%) cases. While no cases of stillbirth, birth injury, birth defects or IUFD were found in our sample. Moreover, we found that those who were obese class 1 are more liable to frequent admission to NICU, and poor Apgar scores. We also noticed they had the higher numbers of macrosomic neonates. Similarly, those with high levels of TSH had more neonates who had poor Apgar scores.

**Table 3:** The neonatal outcomes regarding the sample TSH: Thyroid stimulating hormone; FT4: Free T4; NICU: Neonatal intensive care unit.

Variable		TSH Levels			Total
		Low (N = 2)	Normal (N = 42)	High (N = 13)	
Fetal sex (missing = 4)	Male	0 (0.0%)	24 (58.5%)	9 (81.8%)	33 (61.1%)
	Female	2 (100.0%)	17 (41.5%)	2 (28.6%)	21 (38.9%)
Fetal presentation (missing = 16)	Cephalic	2 (100.0%)	25 (59.5%)	10 (76.9%)	37 (64.9%)
	Breach	0 (0.0%)	4 (9.5%)	2 (15.4%)	6 (10.5%)
APGAR 1 min (missing = 2)	Poor score	0 (0.0%)	3 (7.3%)	1 (8.3%)	4 (7.3%)
	Good score	2 (100.0%)	38 (92.7%)	11 (91.7%)	51 (92.7%)
APGAR 5 mins (missing = 2)	Poor score	0 (0.0%)	0 (0.0%)	0 (0.0%)	0 (0.0%)
	Good score	2 (100.0%)	41 (100.0%)	12 (100.0%)	55 (100.0%)
Birth Weight	Low	0 (0.0%)	2 (4.9%)	1 (9.1%)	3 (5.6%)
	Normal	2 (100.0%)	35 (74.5%)	10 (90.7%)	47 (87.0%)
	Macrosomia	0 (0.0%)	4 (9.8%)	0 (0.0%)	4 (7.4%)
Gestational age	Preterm	0 (0.0%)	5 (12.2%)	2 (15.4%)	7 (12.5%)
	Full term	2 (100.0%)	35 (85.4%)	11 (84.6%)	48 (85.7%)
	Post term	0 (0.0%)	1 (2.4%)	0 (0.0%)	1 (1.8%)
Neonatal death		0 (0.0%)	1 (2.4%)	0 (0.0%)	1 (1.8%)
NICU administration		0 (0.0%)	4 (9.5%)	0 (0.0%)	4 (7.0%)

In respect to postpartum complications, Table 4 shows a total of nine (15.8%) cases had vaginal laceration tear, three (5.3%) had perianal laceration 1st degree, two (3.5%) had perianal laceration 2nd degree, and only one (1.8%) case had OSS. Regarding anemia, it was reported among 34 (59.6%) cases, and UTIs among 18 cases (31.6%). Moreover, we had a significant association (P < 0.05) between elevated levels of TSH and risk of developing UTIs.

**Table 4:** Postpartum complications in the study TSH: Thyroid stimulating hormone; FT4: Free T4; OSS: Open surgical site; UTIs: Urinary tract infections.

Variable		TSH Levels			Total
		Low (N = 2)	Normal (N = 42)	High (N = 13)	
Postpartum complications	Vaginal laceration tear	0 (0.0%)	5 (11.9%)	4 (30.8%)	9 (15.8%)
	Perianal laceration 1st	0 (0.0%)	2 (4.8%)	1 (7.7%)	3 (5.3%)
	Perianal laceration 2nd	0 (0.0%)	2 (4.8%)	0 (0.0%)	2 (3.5%)
	OSS	0 (0.0%)	1 (2.4%)	0 (0.0%)	1 (1.8%)
	Anemia	2 (100.0%)	26 (61.9%)	6 (46.2%)	34 (59.6%)
	UTIs	1 (50.0%)	15 (35.7%)	2 (15.4%)	18 (31.6%)

Regarding delivery as shown in Table 5, CS was the mode of delivery among 40 (61.5%) mothers and SVD was the mode among the rest (25, 43.9%). The types of CS were: 13 (40.6%) emergency and 19 (59.4%) elective.

**Table 5:** Mode of delivery in the sample TSH: Thyroid stimulating hormone; FT4: Free T4; SVD: Spontaneous vaginal delivery; CS: Cesarean section.

Variable		TSH Levels			Total
		Low (N = 2)	Normal (N = 42)	High (N = 13)	
Mode of delivery	SVD	0 (0.0%)	18 (42.9%)	7 (53.8%)	25 (43.9%)
	CS	2 (100.0%)	24 (57.1%)	6 (46.2%)	32 (56.1%)
Type of CS	Emergency	1 (50.0%)	10 (23.8%)	2 (15.4%)	13 (40.6%)
	Elective	1 (50.0%)	14 (33.3%)	4 (23.8%)	19 (59.4%)

## Discussion

It has been long recognized and studied how the physiology of the thyroid gland changes during pregnancy resulting in changes in the parameters of the thyroid function tests [15]. Despite a normal echo structure, the thyroid gland characteristically changes to appear with glandular hyperplasia, increased vascularity, and an approximately 30% increased volume [16]. The physiological and anatomical changes may lead to a high suspicion of thyroid abnormalities and eventually may contribute to the increased frequency of screening [11]. However, the adverse effects of hypothyroidism on gestation and neonatal health are still largely unknown or assessed in our region.

In this study, we aimed to assess the adverse maternal and neonatal outcomes among obese pregnant women with hypothyroidism. Since the number of subclinical and overt hypothyroidism cases in our sample were unequal, we assessed the outcomes using maternal TSH levels. The present study found 57.1%, 33.3%, 9.5% pregnant women with high TSH levels for BMI classes 1, 2, 3 respectively. Similarly, another recent study conducted locally in Riyadh showed the high prevalence of hypothyroidism among pregnant women [17]. Not only that, another study also reported a significant association between high TSH concentrations and high BMI categories [18]. In Lebanon, hypothyroidism was reported in 17% of 920 pregnant women, and it was associated with a history of miscarriage and morbid obesity [11]. All in all, the results of this study showed the prevalence of hypothyroidism among obese pregnant women and established the need for further assessment of the adverse outcomes possible to develop during gestation.

In this present study, we assessed the incidence of the complications that developed during or after pregnancy among 57 obese women diagnosed with hypothyroidism at KAUH. The association between high TSH and adverse pregnancy outcomes was observed in our sample. For the maternal outcomes, it was found that high TSH levels positively correlated with preterm labor and the development of gestational diabetes mellitus (GDM). A recent study showed women who delivered premature babies did not have the usual decrease in TSH levels in early pregnancy and another study found women with subclinical hypothyroidism were twice as likely to deliver prematurely compared to subjects whose thyroid function tests were normal [12,19]. Furthermore, a meta-analysis conducted in China showed that hypothyroidism further amplified insulin resistance thereby leading to increased risk of developing GDM [20]. In another study conducted on 508 Pakistani women, 61% of those women with GDM reported high TSH levels compared to 6% of high TSH levels in healthy controls [21]. Concerning the neonatal outcomes, this study revealed that high levels of TSH were negatively correlated with lower gestational age and lower birth weight neonates. Nevertheless, it is noteworthy that four (7.4%) of the neonates were macrosomic where it was more frequent in babies of obese class 1 mothers. One study reported that mothers diagnosed with hypothyroidism gave birth to children with higher birth weight, but on average, the gestational age was lower [22]. Moreover, pregnant women with high TSH levels had more neonates who experienced low Apgar scores. A recent study conducted showed data of significant low Apgar scores at 1 minute and 5 minutes that negatively correlated with maternal TSH [23]. Interestingly, our study revealed that neonates of frequent admission to NICU and poor Apgar scores were higher from pregnant women of class 1 obesity compared to other BMI classes. The cross-sectional study observed prevalence of NICU admissions, lower mean gestational age and higher mean TSH to mothers with hypothyroidism [23]. In regard to postpartum complications, no significant association was found in our sample. Moreover, regarding mode of delivery, caesarean section was observed at a higher rate than SVD among women with elevated TSH levels. However, to the best of our knowledge and to date, no sufficient studies have been conducted locally to assess the association between elevated TSH and postpartum complications.

Maternal hypothyroidism is not associated with a consistent pattern of adverse outcomes during or after pregnancy, however, it still poses a huge risk for both the mother and her offspring [12,24]. A recent study showed thyroid screening in pregnant women and early treatment of subclinical hypothyroidism (SCH) during pregnancy have been reported to improve, reverse, or decrease the impact of various maternal and neonatal outcomes associated with SCH [12]. Nevertheless, screening for subclinical hypothyroidism or TSH levels remains to be non-carried in antenatal settings in Saudi Arabia [12]. Thus, considering the frequency and the adversity of the pre/postpartum outcomes among obese women with hypothyroidism, it is necessary to establish a baseline of TSH levels for enhanced monitoring of the thyroid function during pregnancy. The enhanced monitoring and frequent screening of thyroid test profile could further prevent the negative outcomes and/or reduce their incidence among our patients.

Currently the benefits of treatment with levothyroxine for women with hypothyroidism during pregnancy remain controversial. The results of meta-analysis of randomized control trials revealed that Levothyroxine (LT4) therapy significantly reduced the miscarriage rate, gestational diabetes, and gestational hypertension, but not preeclampsia [25]. Moreover, the study found that LT4 group had fewer preterm deliveries, birth weights <2500 g, deaths, and congenital malformations compared to control group in regards to neonatal outcomes [25]. A recent study showed that LT4 therapy is associated with a reduced risk of low birth weight (LBW) offspring and a low Apgar score among women with subclinical hypothyroidism [26]. Literature review showed conflicting results concerning the effect of therapy on pregnancy outcomes, and whether the treatment is cost-effective or not [27]. However, larger epidemiological studies are needed in Saudi Arabia to evaluate the effects of treating women with hypothyroidism during pregnancy, and many studies emphasize the need for further assessment on this subject due to hypothyroidism prevalence in our region.

This hospital-based retrospective study has been conducted among patients who attended at King Abdulaziz university hospital, and since it is a single center, we cannot generalize our findings among the population of the western region in Saudi Arabia. The study included pregnant women with hypothyroidism who were obese (BMI Classes 1,2,3) in which it narrowed our sample further. However, the observation of negative pregnancy outcomes of women with hypothyroidism remains to be validated by population studies [12,28,29]. Furthermore, we could not determine whether some of the maternal morbidities were actually caused by thyroid due to the retrospective nature of this study.

## Conclusions

In conclusion, obese pregnant women with hypothyroidism are more prone to adverse maternal and neonatal outcomes than normal population. So, running thyroid function test during prenatal and antenatal will help in finding undiscovered cases of hypothyroidism and help those with the disease to avoid undesired pregnancy outcomes. In addition, implanting a comprehensive plan targeting diet and physical activity with a proper health education prior to their pregnancy is going to help them reducing obesity, which is a known risk factor for adverse maternal and neonatal outcomes. This study also adds to the previous literature, that hypothyroidism during pregnancy is related to a tremendous maternal and neonatal risk, such as GDM, PTL, admission to NICU and neonatal death. Finally, we recommend a population-based cohort study to provide a more accurate knowledge about prevalence and impact of hypothyroidism on pregnancy outcomes.
